# Comment on “Clinical outcomes and histologic findings of patients with hepatocellular carcinoma with durable partial response or durable stable disease after receiving atezolizumab plus bevacizumab”^☆^

**DOI:** 10.1016/j.iliver.2024.100130

**Published:** 2024-11-07

**Authors:** Binghua Li, Qiang Wang, Weiwei Hu, Huan Li, Peng Yan, Yajuan Cao, Decai Yu

**Affiliations:** a Division of Hepatobiliary and Transplantation Surgery, Department of General Surgery, Nanjing Drum Tower Hospital, The Affiliated Hospital of Nanjing University Medical School, Nanjing 210008, China; b Department of Clinical Science, Intervention and Technology (CLINTEC), Division of Medical Imaging and Technology, Karolinska Institutet, Stockholm 14186, Sweden; c Center for New Drug Safety Evaluation and Research, State Key Laboratory of Natural Medicines, China Pharmaceutical University, Nanjing 211198, China

We read with great interest the recent article by Shen et al. [1], which provides valuable insights into the outcomes and histopathologic characteristics of hepatocellular carcinoma (HCC) patients treated with atezolizumab plus bevacizumab (Atezo-Bev), particularly those demonstrating a durable partial response (PR) or stable disease (SD). The authors claimed no reliable radiologic features or blood-based biomarker to predict pathologic complete response (PCR) of the residual tumors. We commend the authors for their valuable contributions to this challenging and underserved area of research. However, several issues require further clarification when interpreting and generalizing the findings.

First, there is a notable discrepancy between the populations in the IMbrave150 study and the real-world cohort. The IMbrave150 study included patients with unresectable HCC, of whom 82% were classified as Barcelona Clinic Liver Cancer stage C [2], while the real-world cohort comprised potentially resectable patients, with 56.2% classified as Barcelona Clinic Liver Cancer stage C. Given that the molecular and cellular characteristics of the tumor microenvironment differ significantly between relatively early and late stages [3], it remains uncertain whether conclusions drawn from neoadjuvant-based immunotherapy can be extrapolated to systemic therapies for unresectable HCC.

Second, we noted that the real-world cohort comprised 32 patients from 16 centers (an average of 2 patients per center). While we acknowledge the difficulty and appreciate the authors’ efforts in collecting such valuable cases, collecting data from a large number of small centers could lead to unreliable estimates of treatment effects and raise analytical challenges [4]. Additionally, maintaining consistency in experimental and therapeutic conditions across different centers, such as pathological examination and treatment courses, is inherently challenging in a retrospective study and could introduce bias.

Third, although the study’s definition of “durable” PR and SD is reasonable and pragmatic, given the lack of a standardized definition for durable response in the literature [5], the conclusion that patients with non-durable responses exhibited markedly worse overall survival and progression-free survival seems somewhat self-evident. This is because the duration of PR or SD, before being classified as “durable”, inherently excludes patients who progress earlier, potentially leading to an overestimation of survival outcomes for the durable PR/SD group. Notably, similar results were not reported for the real-world cohort. The conclusion is based on a single dataset and lacks external validation, which may limit the generalizability of the results.

Additionally, the authors presented representative vignette cases and concluded that no reliable radiologic features could predict PCR. While the statement is important, it should be interpreted with caution, as case series without statistical analysis are generally considered to provide a relatively low level of evidence [6]. Furthermore, only one radiologic feature (i.e., enhancement) was considered. Radiomics, which can be considered a “digital biopsy” of the entire tumor, offers a noninvasive, high-throughput, and comprehensive assessment of tumor biology. Radiomics has emerged as a promising and powerful tool to predict therapeutic responses, including to treatments such as lenvatinib combined with anti-programmed death-1 therapy in HCC [7]. Integrating radiomics and machine learning would significantly strengthen the findings in the study.

Finally, the authors concluded, on the basis of the two case reports, that tumor marker normalization was not predictive of PCR. However, the authors presented data only for alpha-fetoprotein (AFP). Other blood-based biomarkers for HCC, such as serum des-gamma-carboxyprothrombin (DCP), were not examined. A previous study reported that 24.1% of patients in the SD and PR groups exhibited discrepancies between AFP and DCP changes during Atezo-Bev treatment [8]. In one PCR case following preoperative Atezo-Bev therapy, an opposite change in biomarker levels was observed during neoadjuvant Atezo-Bev therapy [9]. Therefore, while AFP remains the most widely used biomarker for HCC, other biomarkers, including DCP, may be considered when predicting responses to Atezo-Bev therapy. We recently performed a comprehensive study that aimed to identify predictive transcriptomic biomarkers for Atezo-Bev therapy [10]. Further research is necessary to explore the potential of transcriptomic biomarkers to predict the PCR to Atezo-Bev in HCC.

In conclusion, while the study by Shen et al. provides valuable insights into the clinical and histologic outcomes of HCC patients treated with Atezo-Bev, the findings should be interpreted with caution, particularly regarding radiologic and serological predictors of pathologic response during Atezo-Bev treatment. Future prospective studies with large cohorts, diverse patient populations, and long follow-up periods are essential to validate the findings in this study and enhance our understanding of immunotherapy in HCC. Additionally, there is great potential to further explore the mechanisms driving durable responses to Atezo-Bev therapy and investigate whether radical hepatectomy could offer additional survival benefits for HCC patients achieving PCR after Atezo-Bev conversion. The integration of multi-omics data could play a pivotal role in providing a comprehensive understanding of HCC biology in the context of immunotherapy. Moreover, advancements in artificial intelligence hold promise for developing robust biomarkers based on routine clinical examinations and multi-omics data, enabling more precise and individualized treatment strategies.

## Funding

This work was supported by grants from the 10.13039/501100001809National Natural Science Foundation of China (Nos. 82372834 and 82173129) and the Jiangsu Outstanding 10.13039/100003536Youth Foundation (BK20240119).

## CRediT authorship contribution statement

**Binghua Li:** Writing – review & editing, Writing – original draft, Investigation, Funding acquisition, Conceptualization. **Qiang Wang:** Conceptualization, Investigation, Writing – original draft, Writing – review & editing. **Weiwei Hu:** Writing – review & editing, Investigation, Data curation. **Huan Li:** Writing – review & editing, Investigation, Data curation. **Peng Yan:** Writing – review & editing, Investigation, Data curation. **Yajuan Cao:** Writing – review & editing, Investigation, Data curation. **Decai Yu:** Writing – review & editing, Supervision, Funding acquisition, Conceptualization.

## Data availability statement

Not Applicable.

## Ethics statement

Not Applicable.

## Informed consent

Not Applicable.

## Declaration of competing interest

The authors declare no conflict of interest.
